# Further support for association between GWAS variant for positive emotion and reward systems

**DOI:** 10.1038/tp.2016.289

**Published:** 2017-01-31

**Authors:** T M Lancaster, N Ihssen, L M Brindley, D E J Linden

**Affiliations:** 1 Neuroscience and Mental Health Research Institute, Cardiff University, Cardiff, UK; 2 Cardiff University Brain Research Imaging Centre, School of Psychology, Cardiff University, Cardiff, UK; 3 MRC Centre for Neuropsychiatric Genetics and Genomics, Institute of Psychological Medicine and Clinical Neurosciences, Cardiff School of Medicine, Cardiff University, Cardiff, UK; 4 Department of Psychology, Queen's Campus, Durham University, Durham, UK

## Abstract

A recent genome-wide association study (GWAS) identified a significant single-nucleotide polymorphism (SNP) for trait-positive emotion at rs322931 on chromosome 1, which was also associated with brain activation in the reward system of healthy individuals when observing positive stimuli in a functional magnetic resonance imaging (fMRI) study. In the current study, we aimed to further validate the role of variation at rs322931 in reward processing. Using a similar fMRI approach, we use two paradigms that elicit a strong ventral striatum (VS) blood oxygen-level dependency (BOLD) response in a sample of young, healthy individuals (*N*=82). In the first study we use a similar picture-viewing task to the discovery sample (positive>neutral stimuli) to replicate an effect of the variant on emotion processing. In the second study we use a probabilistic reversal learning procedure to identify reward processing during decision-making under uncertainly (reward>punishment). In a region of interest (ROI) analysis of the bilateral VS, we show that the rs322931 genotype was associated with BOLD in the left VS during the positive>neutral contrast (*P*_ROI-CORRECTED_=0.045) and during the reward>punishment contrast (*P*_ROI-CORRECTED_=0.018), although the effect of passive picture viewing was in the opposite direction from that reported in the discovery sample. These findings suggest that the recently identified GWAS hit may influence positive emotion via individual differences in activity in the key hubs of the brain's reward system. Furthermore, these effects may not be limited to the passive viewing of positive emotional scenes, but may also be observed during dynamic decision-making. This study suggests that future studies of this GWAS locus may yield further insight into the biological mechanisms of psychopathologies characterised by deficits in reward processing and positive emotion.

## Introduction

Genome-wide association studies (GWAS) are helping to uncover the specific biological factors that support personality^1^ and emotion.^2^ These studies suggest that these characteristics strongly load on the genetic susceptibility for psychiatric disorders^2, 3^ and are therefore essential in elucidating the biological mechanisms that underpin psychopathology. A recent GWAS studied individual differences in positive emotional experiences (PE),^4^ identifying a GWAS significant single-nucleotide polymorphism (SNP; rs322931) associated with PE.

Systems biology approaches such as functional magnetic resonance imaging (fMRI) can offer mechanistic explanations, by which genetic loci can influence heritable traits identified via GWAS. The genetic variant was also associated with expression of blood and brain miR-181a, miR181-b and blood oxygen-level dependency (BOLD) in the ventral striatum (VS) and the amygdala while viewing positive scenes compared with neutral scenes.^4^ The authors hypothesise that the loci may increase an individual's propensity to display positive affect through alteration in reward circuitry that supports the positive valuation of stimuli. The GWAS hit situated at rs322931 was further associated with reduced negative affect and increased resilience, suggesting a potential protective effect against environmental stressors and genetic factors that confer risk to psychopathology.

In the current study we aim to further quantify the neural effects of the PE loci using two cognitive paradigms designed to elicit BOLD in the key hubs in the reward system such as the VS. We expand upon the initial association between rs322931 and VS BOLD during the passive viewing of positive stimuli and aim to identify further mechanistic roles of the variant in the brain's reward circuitry and valuation system. The observations from the study may help us to understand the role of the genetic variant in the brain's valuation system in health and clinical traits characterised by a reduction in positive emotion. Using a similar approach as described in the discovery study, we first use a passive viewing paradigm of positive and neutral picture stimuli^5^ to identify potential neural effects of the rs322931 SNP on VS BOLD. Second, we use a probabilistic reversal learning procedure (as previously described^6^), a reliable paradigm to assess reward-related brain activity,^7^ to identify potential neural effects of rs322931 associated with VS BOLD during receipt of reward, compared with punishment.

We hypothesise that the minor allele of rs322391, which has been associated with PE, will be associated with an increased VS BOLD response to (a) positive stimuli (as previously observed) and during (b) the receipt of monetary reward compared with punishment. The findings from the study will elucidate whether the variant has pleiotropic effects on the brains reward system, or whether the effects of the SNP are specific to the viewing of positive stimuli.

## Materials and methods

### Participants

One hundred right-handed Caucasian (of western European descent) volunteers aged 19–47 were recruited from Cardiff University (staff and/or students) for a study involving several MRI, magnetoencephalography and behavioural paradigms. No participants reported any psychiatric illness^8^ or use of psychotropic medication. Informed consent was obtained for all individuals before the study, which was approved by the ethics committee of the School of Psychology, Cardiff University. A sample of *N*=81 (picture viewing) and *N*=82 (reversal learning) participants was included in the final sample after removing individuals with failed quality control of genetic data (*n*=10) or incomplete imaging data (*n*=9/10, respectively; see Table 1 for participant demographics).These samples largely overlapped (one participant completed the picture viewing task that did not complete the reversal learning task, and two participants completed the reversal learning task that did not complete the picture viewing task); however, this did not affect the smallest rs322931 genotype cell (*n*=10, in both cases).

### DNA extraction and genotyping

Genomic DNA was obtained from saliva using Oragene OG-500 (DNA Genotek, Kanata, ON, Canada) saliva kits. Genotyping was performed using custom genotyping arrays (Illumina HumanCoreExome-24 BeadChip), which contain 570 038 genetic variants (Illumina, San Diego, CA, USA). Quality control was implemented in PLINK^9^ to ensure genotypes did not display ambiguous sex, cryptic relatedness up to third-degree relatives by identity of descent or genotyping completeness <97%. We also removed non-European ethnicity admixture detected as outliers in iterative EIGENSTRAT analyses of an LD-pruned data set.^10^ SNPs were excluded where the minor allele frequency was <1%, if the call rate <98% or if the *χ*2-test for Hardy–Weinberg equilibrium had a *P*-value <1e−04. After quality control, rs322931 genotype information was available for all ninety successfully genotyped individuals.

### fMRI experiment 1: emotional picture viewing

Eighty-one participants randomly viewed affectively positive and neutral stimuli taken from the International Affective Picture System (IAPS)^5^ or Internet resources. We included 18 neutral IAPS pictures having a mean normative valence rating of 4.87 (1=very unpleasant, 9=very pleasant) and 9 positive IAPS pictures having a mean normative valence rating of 6.99. Images taken from other resources had been used and validated in a previous study.^11^ Picture categories were comparable with regard to semantic homogeneity and perceptual complexity: neutral pictures showed household objects and positive images depicted appetitive food. Each block lasted 8 s, in which an array consisting of either four random positive or four random neutral images were presented at a rate of 2 s per image. This process was repeated 10 times for each participant. To keep individuals engaged in the task, we included a 1-back monitoring task in which participants had to confirm with a button press each time an image was presented twice in a row within a trial block. For each participant, we embedded four picture repetitions at random positions within the entire sequence of picture viewing blocks. The number of picture repetitions was balanced across picture categories. There were four picture repetitions for each participant, with an equal number of repetitions occurring for each picture category. Participants viewed a total of 40 stimuli per condition. Intertrial intervals were randomly jittered (6–10 s) in order to sample the haemodynamic response at different time points. To keep individuals engaged in the task, participants had to confirm with a button press each time an image was presented twice in a row within a trial block.

### fMRI experiment 2: probabilistic decision-making procedure

Eighty-two participants learned to choose one of two simultaneously presented colours (‘blue' and ‘green‘) by receiving monetary reward for correct choices and monetary punishment for wrong choices (for example, +1 pence (p) for ‘blue' and −1p for ‘green‘). After 7–11 trials, reward/punishment contingencies were reversed so that the previously rewarded colour was now punished and *vice versa*. Participants were instructed to maximise their earnings during the learning session, which consisted of 12 reversal episodes in total (108 choice trials). Within each reversal episode we included either 1 or 2 PE (probabilistic error) trials, in which ‘wrong' feedback was given for correct choices, even though the reward contingencies had not changed. At the start of each choice trial, participants were presented with a response cue consisting of two white frames surrounding the colours and prompting the participants to press the left or right button on a response box to choose one colour. Response feedback (choice outcome) was given subsequently using a centrally presented white ‘smiley' (correct choice) or red ‘frowny' (incorrect choice) face and an earnings' counter changing incrementally by ±1p. In trials following reversal or PE events, that is, in those trials used for fMRI analysis (see below), response cues and feedback stimuli were presented with a jittered duration (cue: 4–8 s, mean 5.5 s; feedback: 0.75 s followed by 3–7 s (mean 4.5 s) intertrial interval). To reduce scanning time, in all other (standard) trials we used fixed and shorter stimulus durations (cue: 2 s, feedback: 0.75 s). ITIs showed the two colours without response cue or feedback and were 0.5 s long after standard trials and between 4 and 8 s (mean 5.5 s) after PEs and reversals. BOLD response analysis focused on brain activation differences as a function choice outcomes (reward>punishment) in post PE and post-reversal trials. We selected those trials for analysis as they yielded a comparatively balanced number of rewards or/and punishments (correct versus incorrect choices) compared with standard trials (which were disproportionally more rewarded than punished). We have previously published fMRI data from this experiment in relation to polygenic risk for schizophrenia.^6^

### Behavioural data analysis

Overall learning performance was assessed as the accumulated earnings across all 108 choice trials. We also calculated trial-based average accuracies (% choices corresponding to the correct colour of each reversal episode) for the trials directly following PE and reversal events (post PE and post-reversal trials) for each participant. Post PE and post-reversal accuracy scores allowed us to measure impulsive choice behaviour (high switch rates/low accuracies after PEs) and perseverative tendencies (low switch rates/low accuracies after reversals).

### Image acquisition

Gradient echoplanar imaging data were acquired for each subject using a 3 T GT HDx system with an eight-channel receiver at Cardiff University Brain Research Imaging Centre, School of Psychology, Cardiff University (parameters: 35 slices, slice thickness; 3 mm/1 mm gap, acquisition matrix; 64 × 64; field of view (FOV); 220 mm, repetition time (TR) 2000 ms, echo time (TE) 35 ms, flip angle 90°, acceleration (ASSET) factor; 2). High-resolution three-dimensional T1-weighted images were also acquired using a three-dimensional fast spoiled gradient echo sequence with 172 contiguous sagittal slices of 1 mm thickness (TR 7.9s, TE 3.0 ms, Inversion time (TI) 450 ms, flip angle 20°, FOV 256 × 256 × 176 mm, matrix size 256 × 256 × 192 to yield 1 mm isotropic voxel resolution images). All functional images were first motion-scrubbed, where TRs with a framewise displacement >0.9 were removed, as previously recommended.^12^

### Image processing

Image processing and statistical analyses were conducted using statistical parametric mapping methods as implemented in the FMRI Expert Analysis Tool (FEAT, Version 5.98, part of FMRIB's Software Library, www.fmrib.ox.ac.uk/fsl). The following pre-statistics processing was applied; motion correction using MCFLIRT;^13^ slice-timing correction using Fourier-space time series phase-shifting; non-brain removal using BET (Brain Extraction Tool);^14^ spatial smoothing using a Gaussian kernel of FWHM 5 mm; grand-mean intensity normalisation of the entire 4D data set by a single multiplicative factor; and high-pass temporal filtering (Gaussian-weighted least-squares straight line fitting, with sigma=50.0 s). Registration to high-resolution structural (single-subject general linear model (GLM)) and standard space (group-level GLM) images was carried out using FLIRT.^13^ Time series analysis was carried out using FMRIB's Improved Linear Model with local autocorrelation correction.^15^ To further correct for any potential movement confounds, motion regressors estimated via MCFLIRT and scrubbed TRs were added as covariates of no interest to the design matrix. Group-level analysis was carried out using FLAME (FMRIB's Local Analysis of Mixed Effects).^16^

### fMRI analysis

To index neural responses to positive emotional stimuli in experiment 1, BOLD signal changes were regressed by task predictor functions (positively affective stimuli > neutral stimuli) convolved with a canonical haemodynamic response function. Predictor time courses were locked to the onset of feedback stimuli, with a fixed duration of 3750 ms, which corresponded to the earliest possible start of the next choice trial, and convolved with a canonical haemodynamic response function. For each subject, statistical contrast images reward>punishment were obtained.

### fMRI analysis: group statistics

We ran multiple regression using the combined first-level contrasts (positive>neutral and reward>punishment contrast images) for each subject covarying for the rs322931 genotype and potential confounds (age and sex). The rs322931 genotype status was entered into the multiple regression as the number of T alleles (the allele associated with increased positive emotion^4^) possessed by each participant (0, 1 or 2); therefore, a positive association would represent a T allele dose-dependent increase in BOLD. We explored the (a) group-level contrasts (one sample *t*-tests) and (b) rs322931 genotype effects (multiple regressions) in the VS region of interest (ROI), defined as the bilateral accumbens in the Harvard–Oxford Subcortical Structural Atlas. The familywise error rate was controlled in all cases with nonparametric permutation testing (5000 permutations) and threshold-free cluster enhancement, which effectively controls for multiple comparisons, compared with cluster extent thresholding.^17, 18^

## Results

### Demographics and behaviour

The rs322931 was not over-represented in either gender or was associated with age in either fMRI experiment. There were no associations between rs322931 genotype and performance during the reversal learning procedure (see Table 1 for demographic details). One-way analysis of variance confirmed that there were no rs322931 genotype differences in the number of reward/punishment events presented to the participants across the reversal learning task (F_2,81_=0.408, *P*=0.666).

### Group-level contrasts

**Figure 1:**
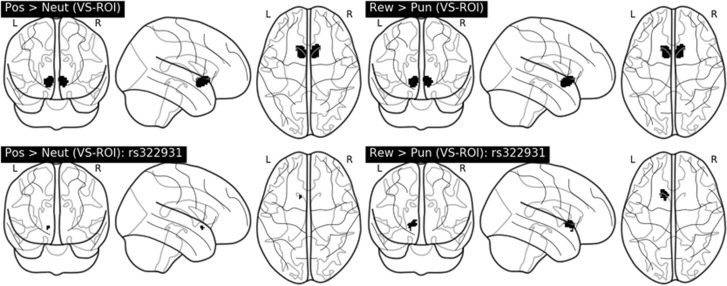
Top—left: one sample *t*-test for positive>neutral stimuli. Top—right: one sample *t*-test for reward>punishment during periods of uncertainty during reversal learning. Bottom—left: effect of rs322931 genotype in the positive>emotion contrast. Bottom—right: effect of rs322931 genotype in the reward>punishment contrast. All active voxels (in black) are corrected for the familywise error (*P*_ROI-CORRECTED_<0.05) across the ventral striatum (VS) using threshold-free cluster enhancement. ROI, region of interest.

We conducted two one-sample *t*-tests to ascertain that the functional networks were recruiting the VS when comparing (a) positive>neutral stimuli and (b) reward>punishment. After correcting for the familywise error rate using threshold-free cluster enhancement across the VS (P_ROI-CORRCETED_<0.05), we found widespread activation in the VS for both contrasts. Specifically, in the positive>emotion contrast, we observed two clusters: *k*=247 (*x*=−8, *y*=6, *z*=−14), *k*=206 (*x*=8, *y*=16, *z*=0), *P*_ROI-CORRECTED_=<0.001 in both cases. In the reward>punishment contrast, we observed *k*=241(*x*=−8, *y*=6, *z*=−14), *k*=219 (*x*=12, *y*=8, *z*=−14), *P*_ROI-CORRECTED_<0.001 in both cases (see Figure 1, top right and left, respectively).

### rs322931 genotype effects

Within the Positive > Neutral Stimuli contrast, we observed a negative association between rs322931 genotype and BOLD within a cluster in the left VS, k=9 (x=- 12, y=10, z=−8), P_ROI-CORRECTED_=0.045 (corresponding to lower activation for the T allele). There were no rs322931 genotype related differences that survived correction for multiple comparisons across the whole brain (P_CORRECTED_ >0.26). There were no positive associations rs322931 and BOLD in the VS or across the whole brain (P_CORRECTED_ > 0.5, in both cases). For the Reward > Punishment contrast, we observed a positive association between rs322931 genotype and BOLD within a cluster in the left VS, *k*=113 (x=−14, y=14, z=−4), P_ROI-CORRECTED_=0.018 (see Figure 1, bottom right and left, respectively) (corresponding to higher activation for the T allele). There were no negative associations rs322931 and BOLD in the VS or across the whole brain (P_CORRECTED_ > 0.5, in both cases).

### Associations between VS BOLD and reversal learning behaviour

There were no significant correlations between the parameters estimates from the rs322931 linked clusters (that survived correction for family wise error across the VS) and behavioural performance during reversal learning (*P*>0.05, in all cases). Modelling behavioural performance during the reversal learning paradigm did not affect the association between VS BOLD and rs322931 genotype (P_ROI-CORRECTED_=0.025).

## Discussion

We found rs322931 genotype-related differences in VS BOLD during the processing of rewarding stimuli in a sample of healthy individuals. However, unlike the discovery study, we found that the minor allele (T) at rs322931 was associated with reduced VS BOLD when viewing positively valence stimuli (compared with neutral stimuli), as opposed to the positive association between rs322931 T allele and VS BOLD described in the discovery sample. This discrepancy may be attributed to differences in the positively affective stimuli between the discovery sample and our study, as we showed positive affective pictures of appetitive food rather than complex visual scenery involving facial information. Furthermore, in the present study, task compliance/attentiveness was monitored with a 1-back design, whereas Wingo *et al.*^4^ employed a rating scale. There were also sample differences in age, education levels, gender and ethnicity that may limit comparisons between these findings. For example, several lines of evidence suggest that the VS response to rewarding stimuli changes across adulthood,^19, 20, 21^ and our sample, was considerably younger (mean age: 24±3.6) than the fMRI sample in the discovery sample (mean age: 40±12.8). Consistent with our broader hypothesis, we also show that increased VS BOLD was associated with the minor allele of rs322931 during the receipt of rewarding (compared to punishing) stimuli. The observation suggests that rs322931 may have pleotropic effects on the reward system that underpin its association with increased positive emotion, increased resilience and reduced negative affect.

Several studies suggest that VS BOLD is altered in psychiatric disorders^22-24 [Bibr bib24]^ and in individuals with increased genetic risk to psychopathology,^6, 25, 26^ suggesting that altered VS BOLD may be a neural antecedent that confers risk to psychopathology. Striatal BOLD for rewarding stimuli during reversal learning has also been associated with individual differences in extrinsic and intrinsic motivation,^27^ suggesting that BOLD during this condition may reflect individual differences in orientation towards incentivised stimuli. Across a wider range of paradigms that elicit VS BOLD, individual differences in this key hub within reward circuitry may also relate to individual differences in traits such as reward sensitivity^28, 29^ and clinical symptoms such as anhedonia, avolition and apathy.^30, 31, 32, 33, 34, 35^ Together, these studies suggest that risk and resilience to psychopathology may be (in part) related to VS BOLD and related behavioural manifestations.

We suggest that our findings are regarded with the following considerations. First, the results are based on a limited sample size (we had ~37–53% power in the current study) and will need to be replicated in larger, independent cohorts. Second, we did not have a behavioural measure of PE. These would have been useful in validating behavioural differences between rs322931 genotypes in the sample and exploring putative mediation models between genotype, VS BOLD and behaviour. Last, the direction of association between BOLD for positive stimuli and rs322931 genotype was in the opposing direction (attenuated BOLD was associated with positive emotion VS BOLD in our study) as anticipated and previously observed. Furthermore, it must be appreciated that the VS BOLD response to reward is not only shaped by genetic effects.^25^ This response may also be influenced by factors such as maternal smoking behaviour,^36^ environmental stressors and early-life trauma.^37, 38, 39^ We did not assay these environmental stressors and acknowledge that our findings should be considered with these confounds in mind.

In conclusion, we provide independent evidence that the GWAS significant locus at rs322931 may influence positive emotion via individual differences in VS BOLD response to rewarding stimuli. We suggest that this effect is not limited to the passive viewing of positive emotional scenes but can also be observed during reward feedback when individuals make decisions during periods of uncertainty. These findings add to a growing body of work, suggesting that individual differences in brain reward systems are heritable^40^ and may shape an individual's risk or resilience to environmental stressors and related psychopathology.^37^ Elucidating the genetic architecture that underpins traits such as PE may help us to understand mechanisms of risk and resilience for psychopathology across nonclinical populations. Translating the findings of GWAS loci using techniques such as fMRI are essential in establishing links between genetics and transdiagnostic phenotypes such as those indexed by positive valence systems by the Research Domain Criteria.^32^

## Figures and Tables

**Table 1:** Demographic and behavioural data for final fMRI samples

	*rs322931*			
	*TT*	*CT*	*CC*	P
*Exp. 1*	*10*	*25*	*46*	
Age	25.10 (4.36)	23.08 (3.06)	24.09 (3.59)	0.276^a^
Gender	M=4, F=6	M=12, F=13	M=16, M=30	0.553 _χ_^2^
				
*Exp. 2*	*10*	*26*	*46*	
Age	25.10 (4.36)	23.42 (3.47)	24.07 (3.61)	0.461^a^
Gender	M=4, F=6	M=12, F=14	M=17, M=29	0.747_χ_^2^
				
*Reward task performance*				
Accuracy first reversal (%)	62.73 (21.62)	63.29 (21.89)	71.74 (18.41)	0.158^a^
Accuracy first PE (%)	55.83 (32.17)	63.46 (26.99)	61.05 (30.58)	0.787^a^
Accuracy second PE (%)	42.5 (31.29)	33.65 (27.33)	33.15 (29.37)	0.644^a^
Total earnings (pence)	0.18 (0.21)	0.22 (0.15)	0.24 (0.16)	0.501^a^

Abbreviations: Exp., experiment; F, female; fMRI, functional magnetic resonance imaging; M, male; PE, positive emotional experience.

Exp.1=positive>neutral contrast; reward>punishment. Exp. 2=reward>punishment contrast. _χ_^2^ denotes rs322931 genotype differences were tested with *χ*^2^-test.

a rs322831 genotype differences were examined with one-way analysis of variance.
